# Dermatomyositis With Dual Positive Anti-melanoma Differentiation-Associated Gene 5 and Anti-Sjogren Syndrome-Related Antigen 52 kD Antibodies: A Case Report and Literature Review on Clinical Characteristics

**DOI:** 10.7759/cureus.34235

**Published:** 2023-01-26

**Authors:** Guru Prasad Parthiban, Sowbharnika Arivazhagan, Shiva Charan Anaji, Aaron Williams

**Affiliations:** 1 Internal Medicine, Baton Rouge General, Baton Rouge, USA

**Keywords:** idiopathic inflammatory myopathies, anti-ssa antibody, rapid progressive interstitial lung disease, dermatomyositis, anti-mda5 amyopathic dermatomyositis

## Abstract

Anti-melanoma differentiation-associated gene 5 (MDA 5) is one of the subtypes of dermatomyositis associated with rapidly progressive lung disease. MDA 5 carries a high mortality risk due to respiratory failure. The exact pathophysiology is unclear, but it is linked to genetic predisposition and viral triggers with the associated innate response and cytokine production like interleukins IL-1,6,18, tumor necrosis factor-alpha, and interferons. It is usually treated with anti-cytokines, high-dose steroids, immunosuppressants, and plasma exchange. Due to the atypical presentation and rapidity of the disease course, the diagnosis is often delayed. We report a 39-year-old female presenting with rapidly progressive lung disease secondary to an aggressive form of dermatomyositis.

## Introduction

Dermatomyositis (DM) is a rare auto-immune disease belonging to the idiopathic inflammatory myopathy (IIM) group. IIM has a reported incidence of 0.1 to 1 per 100,000 person-years and a prevalence of 0.55 to 0.60 per 100,000 person-years in the United States [1]. Dermatomyositis is marked by muscle weakness, skin rashes, and widespread systemic organ involvement. The most common cause of early mortality and high morbidity is interstitial lung disease leading to respiratory failure. Other essential features include arrhythmia, heart failure, dysphagia, and chest wall weakness. There are various subtypes of DM, including classic DM (CDM), clinically amyopathic DM (CADM), cancer-associated DM, and anti-melanoma differentiation-associated gene 5 (MDA5) DM. Anti-MDA 5 is an uncommon subtype associated with rapidly progressive interstitial lung disease (RPILD) without myositis features. RPILD is a type of ILD where the disease exacerbation occurs within four weeks of the onset of symptoms. Dual expression of anti-SSA 52 kD and anti-MDA 5 antibodies indicates an aggressive course with high mortality. Due to the acuity of disease presentation with predominant lung involvement, diagnosis may often be delayed. To avoid delay, it is crucial to differentiate the clinical features from other acute pulmonary disease causes like drug or toxin-induced pneumonitis and infections like viral pneumonitis. Testing for autoantibodies like myositis specific (MSA) and myositis associated (MAA) can be helpful in further identification of clinical subtypes and predicting the response to treatment. We hereby discuss an atypical disease presentation of dermatomyositis with positive MDA5 and anti-SSA, an aggressive form of dermatomyositis.

## Case presentation

**Figure 1:**
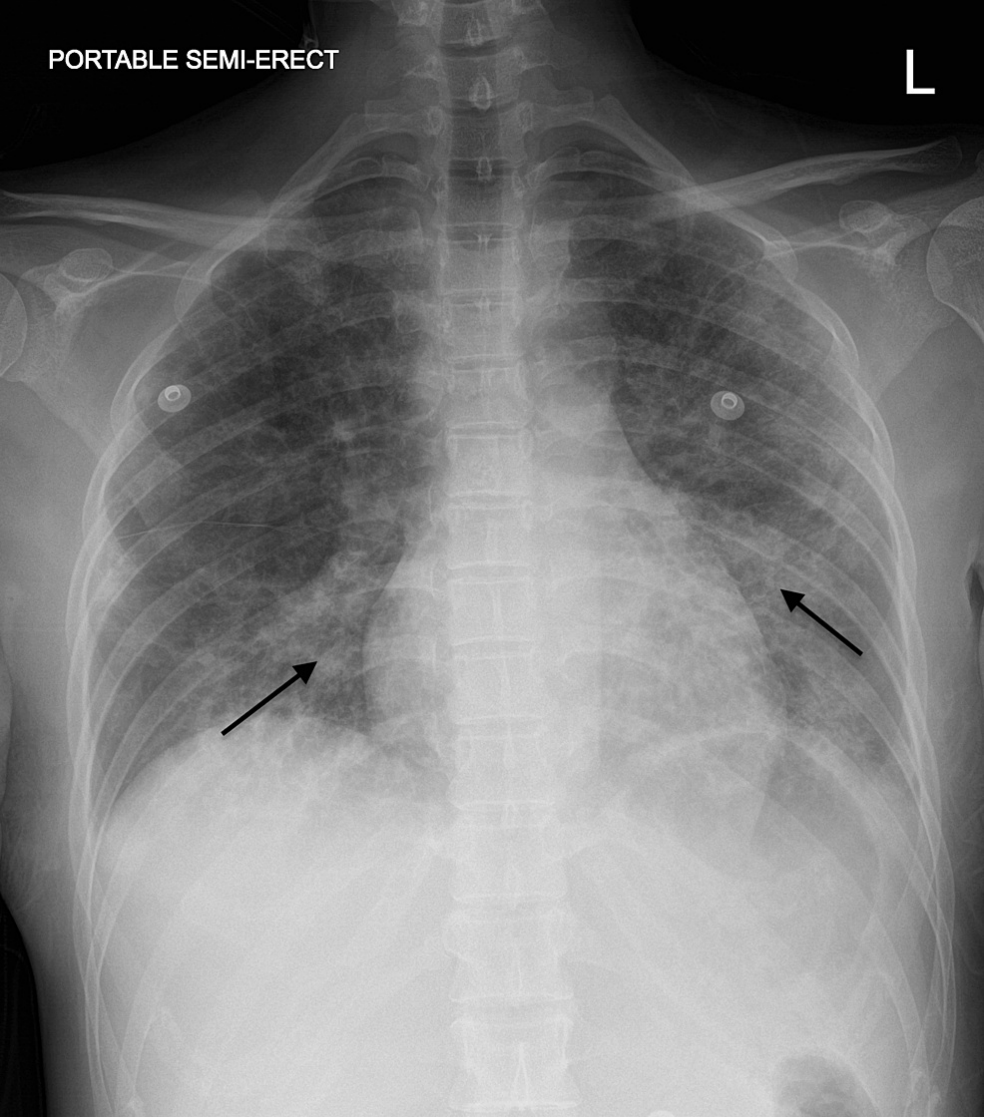
The chest X-ray revealed bilateral interstitial infiltrates (see arrows)

**Figure 2:**
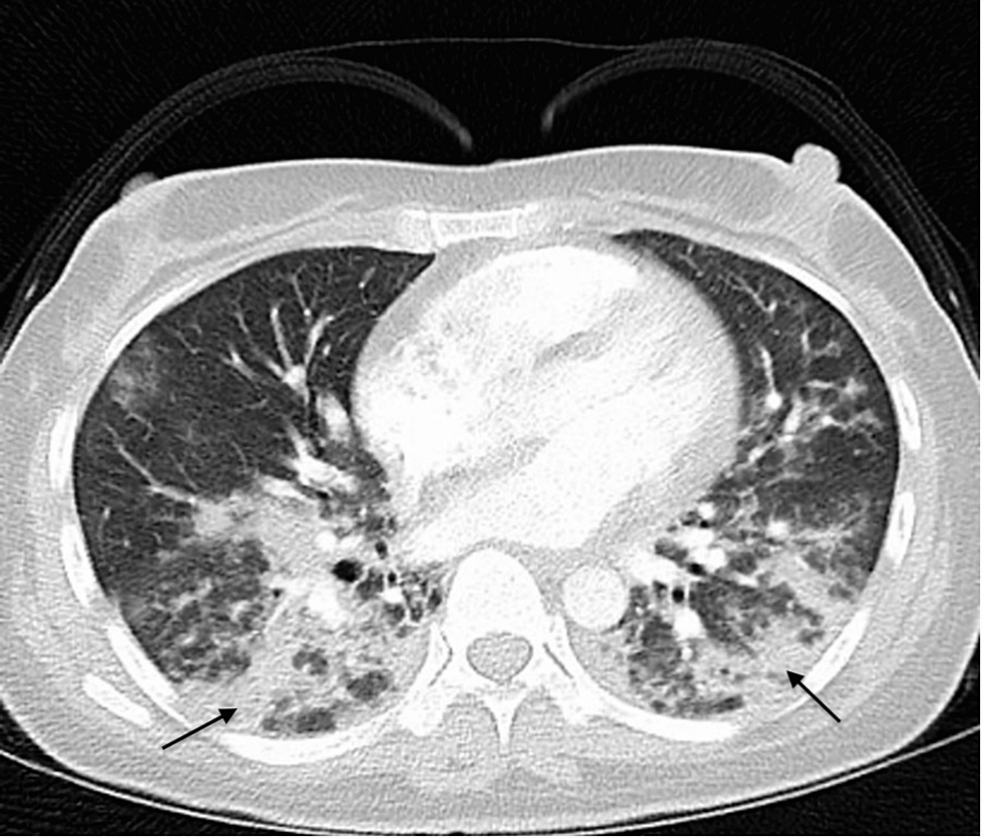
The CT of the chest was significant for extensive ground glass/alveolar opacities (see arrows).

The patient is a 39-year-old female with no significant medical history who presented with two weeks of gradual shortness of breath and intermittent cough with whitish sputum production associated with subjective fever and pleuritic chest pain. She immigrated to the US about 12 years ago. She also endorsed a four-week history of progressive polyarthralgia, especially in her hands, shoulders, and knees, and fatigue for two months. She denied hemoptysis, orthopnea, paroxysmal nocturnal dyspnea, lower extremity edema, or weight loss. The patient denied any history of smoking or illicit drug use. On presentation to the emergency department, her oxygen level via pulse oximetry was 84% on room air, which improved to 97% on two liters of oxygen via nasal cannula. Physical examination was significant for auscultating bilateral basal crackles in the lungs. The skin examination revealed dry skin with minimal scaling noted in both hands. The chest X-ray revealed bilateral interstitial infiltrates, and the CT scan was significant for extensive ground glass/alveolar opacities (Figures 1-2). The patient was initiated on antibiotics for community-acquired pneumonia. A complete respiratory panel with COVID-19 antigen was negative. Further rheumatological workup revealed positive anti-nuclear antibodies (ANA) and mildly positive anti-SSA 52 kD antibody. Notably, HIV and hepatitis panels were negative. Creatinine kinase (CK) was within normal limits, but serum aldolase was elevated. The remaining workup, including rheumatoid factor, anti-SSB, anti-ribonucleoprotein, anti-Smith, anti-double-stranded DNA, cytoplasmic-anti-neutrophil cytoplasmic antibody (ANCA), perinuclear-ANCA was negative. The patient was then initiated on prednisone 40mg daily with mild improvement in her muscle pain. Over the course of two weeks, she had a worsening respiratory status with increasing oxygen requirements. She eventually underwent broncho-alveolar lavage with a lung biopsy for definitive answers. It revealed acute lung injury with diffuse alveolar damage and focal areas of organizing pneumonia. The procedure was complicated by pneumothorax necessitating chest tube placement, and she was subsequently intubated.

The differential diagnosis at this point was anti-synthetase syndrome (aldolase was elevated with a normal CK pointing towards an inflammatory myopathy that affects the perimysium) vs. acute interstitial pneumonia, given the rapidity of the course. Then, the patient was initiated on high-dose corticosteroids with methylprednisolone 60mg every 12 hours. Myomarker 3 profile/myositis panel result came back later in the course negative for anti-synthetase antibodies (anti-Jo1, anti-PL7, anti-PL12, anti-OJ, anti-EJ) but positive for anti-MDA 5 and anti-SSA 52 kD antibodies, indicating an aggressive form of dermatomyositis. The patient was then started on plasma exchange with plans to complete five exchanges. With no improvement following the third exchange, she received cyclophosphamide 500 mg IV once and rituximab 1g IV once. The patient was also started on baricitinib 2mg daily, as there was evidence of survival benefits with Janus kinase (JAK) inhibitors. However, eventually, she had a vasopressor refractory shock and developed severe metabolic acidosis requiring emergent renal replacement therapy. Despite these efforts, the patient passed away due to multiorgan failure.

## Discussion

Dermatomyositis (DM) is a rare autoimmune disease involving the skin and muscles. Auto-antibodies involved are distinguished into myositis-associated (MAA) and myositis-specific (MSA) [2]. There are various subtypes, including classic DM (CDM), clinically amyopathic DM (CADM), cancer-associated DM, and anti-melanoma differentiation-associated gene 5 (MDA5) DM [3]. Among the different subtypes of DM, the prevalence of anti-MDA5 DM ranges from 7-60%, with a higher prevalence in Asians (11-60%) than in the Caucasian (7-16%) population [4]. The outcomes also depend on ethnicity, with poorer outcomes in Japanese but excellent outcomes in the Caucasian population [5]. Our patient was dually positive for anti-MDA5 and anti-SSA 52 kD antibodies, which has an aggressive clinical course and poorer outcomes. These two antibodies have a strong association with juvenile myositis as well [6]. In a study by Xu et al., a cohort of 83 patients with anti-MDA5-positive DM was followed. The clinical features and 24-month survival were compared between anti-MDA5-positive patients with and without anti-SSA 52 kD antibodies [7]. Dual antibody-positive patients had a significantly higher incidence of rapidly progressive interstitial lung disease and cutaneous ulcerations. Moreover, the dual antibody-positive group's 24-month survival rate was significantly reduced [7]. In another study by Xing et al., ILD in patients who had both anti-SSA 52 and anti-MDA5 was found to be worse, and anti-SSA 52 was found to be an independent risk factor for ILD in DM [8].

Some skin manifestations unique to anti-MDA5-positive DM include skin ulceration, necrosis, palmar papules, and digital ischemia. Skin ulcers are usually found over the fingertips, nail folds, and extensor surfaces. Our patient had minimal scaling noted in her fingertips but no ulcers or ischemia. Pneumothorax and pneumomediastinum are fatal complications associated with anti-MDA5 ILD and associated with worse survival [3]. Tendon rupture and periorbital edema are due to vasculopathy and were found more among the individuals who tested positive for both anti-SSA 52 and anti-MDA5 [9]. In addition, a study by Fiorentino et al. showed that diffuse alopecia, mechanic hands, and elbow/knee erythema (Gottron sign) were predominant in the anti-MDA5 positive compared to the anti-MDA5 negative population. Moreover, the classic skin signs of DM, such as Gottron papules and heliotrope rash, were not associated with MDA5 antibodies [10].

Activation of monocytes and macrophages is involved in the pathophysiology of anti-MDA5 positive DM as indicated by elevated serum interleukins (IL-6,10 and 18) and macrophage colony-stimulating factor (M-CSF), more significant in anti-MDA5 positive than in negative patients. Among these cytokines, serum IL-18 can be used to assess the response to the treatment of ILD. Hyperferritinemia predicts a poor prognosis, especially if the ferritin levels are >1600ng/mL [11]. It has been reported that ground glass opacity and consolidation in a subpleural, lower lung field distribution is typical in anti-MDA5 antibody-positive patients. In contrast, peri-bronchovascular distribution is seen in anti-MDA5 antibody-negative patients [9]. Our patient had extensive ground glass/alveolar opacities and mild reticular interstitial changes throughout the lungs, with relative sparing of the anterior portions of the bilateral upper lobes and middle lobes (Figure 1). Anti-MDA5 antibodies are commonly associated with clinically amyopathic DM, which explains the lower frequency of CK elevation in the anti-MDA5 positive population [12]. The classic histopathological picture of the anti-MDA5 positive population includes the absence of peri-fascicular fiber atrophy and characteristic vasculopathy with overall minimal inflammatory changes [13].

An effective treatment regimen includes combination immunosuppressive therapy with high-dose steroids, oral cyclosporine, and intravenous cyclophosphamide pulse [9]. High HRCT (high-resolution computed tomography) scores and high ferritin levels at baseline indicate poor prognosis and poor response to immunosuppressive therapy [14]. In a multicenter prospective study by Tsuji et al., 29 patients with MDA5 DM with RPILD were administered a combination of steroids (prednisolone 1mg/kg/day), cyclophosphamide (IV CYC - initiated at 500 mg/m2 of body surface area biweekly, then gradually increased to 1,000 mg/m2 of body surface area) and tacrolimus (dose adjusted to maintain 12-hour blood trough levels within the range of 10-12 ng/ml) up front. These 29 patients were followed up regularly at 4, 8, 12, 16, 24, and 52 weeks after the initiation of treatment for lung imaging, blood, and urine analysis. This data was compared to historical data where the patients received step-up immunosuppression (initially on prednisolone, with other immunosuppressants added stepwise). The steroid and other immunosuppressant dosages were comparable between the two groups. The six-month and 12-month survival rates were better for the combination group than the step-up group (89% vs. 33% and 85% vs. 33%, respectively) [3]. JAK inhibitors have recently gained popularity, and tofacitinib has shown an increase in survival and a decrease in ferritin levels and CT scores [15]. Plasma exchange can also be used as it replaces the patient’s plasma with that of a healthy person. To determine whether plasma exchange can be used therapeutically; predictive markers such as serum ferritin levels, age, and the severity of pulmonary dysfunction need to be assessed [14]. Short-term plasma exchange has been shown to improve patients’ respiratory and skin manifestations with a significant reduction in anti-MDA5 and ferritin levels and has a favorable prognosis [11]. Plasma exchange can be an effective salvage therapy for resistant anti-MDA5 patients who have failed combination immunosuppressants. Direct hemoperfusion using a polymyxin B-immobilized fiber column (PMX-DHP) has been shown to decrease endotoxins and inflammatory cytokines and is effective in RPILD with CADM [11].

## Conclusions

To conclude, several unique features of anti-MDA5-positive DM should be recognized earlier, given its strong association with RPILD and potentially adverse survival outcomes. Moreover, several studies including this report show that when patients co-express anti-MDA5 and anti-SSA 52 kD antibodies, the mortality rate significantly increases. As most of these patients rapidly deteriorate, it is important to involve specialists early in the course and start treatment without delay.
